# Studying the Effects of Transcranial Direct-Current Stimulation in Stroke Recovery Using Magnetic Resonance Imaging

**DOI:** 10.3389/fnhum.2013.00857

**Published:** 2013-12-12

**Authors:** Charlotte J. Stagg, Heidi Johansen-Berg

**Affiliations:** ^1^Nuffield Department of Clinical Neurosciences, Oxford Centre for Functional MRI of the Brain (FMRIB), John Radcliffe Hospital, University of Oxford, Oxford, UK

**Keywords:** transcranial direct-current stimulation, stroke recovery, MRI, humans, MRS spectroscopy

## Abstract

Transcranial direct-current stimulation (tDCS) is showing increasing promise as an adjunct therapy in stroke rehabilitation. However questions still remain concerning its mechanisms of action, which currently limit its potential. Magnetic resonance (MR) techniques are increasingly being applied to understand the neural effects of tDCS. Here, we review the MR evidence supporting the use of tDCS to aid recovery after stroke and discuss the important open questions that remain.

## Introduction

Transcranial direct-current stimulation (tDCS) is a non-invasive neuromodulatory technique that has been increasingly investigated as a putative therapy for a wide range of neurological and psychiatric conditions including stroke (Hummel and Cohen, 2006), pain (Fregni et al., 2006b), and depression (Kalu et al., 2012). However, despite some promising clinical effects in stroke patients (see Bastani and Jaberzadeh, 2012; Butler et al., 2013 for recent systematic reviews), progress in optimizing tDCS for therapeutic use has been hindered by a limited understanding of the effects of tDCS on the brain. Non-invasive magnetic resonance (MR) approaches, including functional magnetic resonance imaging (fMRI) and magnetic resonance spectroscopy (MRS), have clear advantages as techniques for investigating underlying changes induced by tDCS within the brain.

Here we review the evidence provided by MR studies as to the cortical and subcortical effects of tDCS in the context of the motor system, and how these findings can inform us about optimizing tDCS as a therapeutic tool. We focus on application of tDCS in the chronic stages of recovery of function after a stroke (usually defined as >6 months post event). We aim to place the results from imaging studies within a theoretical framework for stroke recovery, and summarize the evidence supporting that framework as well as the questions that remain currently unanswered.

### Overview of MR methods

#### Functional MRI

##### Task-based functional MRI

Functional magnetic resonance imaging is a versatile and non-invasive tool that can be used to inform our understanding of how tDCS can modulate activity within the brain. The majority of the studies discussed in this review rely on quantification of the blood oxygen level dependent (BOLD) contrast, the most widely used fMRI technique. The BOLD signal arises from the observation that oxygenated and deoxygenated hemoglobin (DeoxyHb) have different magnetic properties, such that oxygenated hemoglobin (OxyHb) is diamagnetic, meaning it has little effect on the magnetic field, whereas DeoxyHb is paramagnetic, meaning that it has a significant interaction with the applied magnetic field. Therefore, if the ratio of OxyHb:DeoxyHb changes within a localized region of tissue as a result of local neuronal activity, then this can be detected using BOLD fMRI. However, the precise relationship between changes in neuronal activity and a detectable change in the BOLD signal is complex and not yet fully understood (Logothetis, 2008).

##### Resting state fMRI

Resting state fMRI examines low frequency fluctuations (generally <0.1 Hz) in brain activity which occur in the absence of task performance (Biswal et al., 1995). Temporally correlated fluctuations in resting fMRI signal between regions are commonly considered to reflect underlying functional connectivity, with increased temporal correlation commonly taken to reflect increased connectivity (Fox and Raichle, 2007; Snyder and Raichle, 2012).

Resting state fMRI is a potentially powerful approach in studying stroke recovery as it removes the confound of task performance (Fornito et al., 2013), but it is not yet clear how changes in resting state connectivity can be related to function. In addition, there is some way to go to make the results of these studies accessible and interpretable to a wider audience (Johansen-Berg, 2013).

#### Structural imaging

##### Gray matter imaging

Structural changes in the brain related to plasticity can be assessed via T1-weighted and T2-weighted MR imaging (Zatorre et al., 2012). In particular, voxel-based morphometry (VBM) of T1-weighted images, has been shown to be a sensitive marker for the degree of recovery seen after stroke (Gauthier et al., 2008, 2012). Although the biological basis of such brain changes remains to be completely elucidated (Zatorre et al., 2012), structural imaging has the potential to be of value in studying the use of tDCS in chronic stroke. As well as enabling study of structural changes induced by tDCS, it has a potential importance as a predictor for subsequent tDCS-induced functional improvement after stroke. Further, the combination of T1-weighted and T2-weighted images are showing promise for individually modeling current flow in stroke patients, an important next step for the optimization of tDCS in this population (Datta et al., 2011, 2012; Neuling et al., 2012).

##### Diffusion tensor imaging

Diffusion tensor imaging (DTI) is an MRI technique that is sensitive to the self-diffusion of water molecules and has been widely used in the stroke rehabilitation literature to investigate tissue microstructure within the major white matter tracts. One metric in particular, fractional anisotropy (FA), is sensitive to subtle decreases in tract integrity although it is influenced by a number of factors such as membrane and myelin integrity as well as fiber density, and therefore the biological basis of the measure is somewhat complex (Beaulieu, 2009). Despite this relative lack of specificity in the measure, FA has been widely used to study recovery after stroke and, as a non-invasive marker of white matter integrity, has been related to functional outcome after stroke both in the chronic (Stinear et al., 2007; Lindenberg et al., 2010a) and sub-acute (Puig et al., 2010) stages of recovery.

#### Magnetic resonance spectroscopy

Magnetic resonance spectroscopy allows the quantification of neurochemicals within a localized region of tissue, and technical advances now mean that accurate measurement of the levels of both GABA and glutamate are possible within a clinically feasible timescale. MRS measures the total amount of a given neurochemical within a region of interest and therefore directly relating MRS-acquired measures of neurotransmitters to synaptic activity can be problematic. However, with mounting evidence as to the physiological basis of the MRS-acquired signals for both GABA and glutamate (Petroff and Rothman, 1998; Stagg et al., 2011b) MRS is becoming an increasingly useful tool in studying physiological changes induced to tDCS (Stagg, 2013). A recent tDCS study in chronic stroke patients by O’Shea et al. (2013) demonstrated that GABA levels within the ipsilesional M1 predicted a patient’s response to anodal tDCS to that region, such that higher initial GABA levels were linked to greater behavioral gains with stimulation.

### Specific considerations for the use of MR to study stroke recovery and transcranial stimulation

Although BOLD fMRI is the most commonly used fMRI technique for studying the modulation of activity in response to transcranial stimulation protocols, it is important to note some caveats that should be applied when interpreting the results of these studies.

Increases or decreases in BOLD signal under the electrode, where the applied current density is highest (Miranda et al., 2006), may be driven by tDCS effects directly on the small vessels within the cortex, as seen in skin vessels following anodal tDCS (Durand et al., 2002); or by tDCS-related modulation of neurovascular coupling, rather than by tDCS-induced changes in neuronal firing directly. It is less likely, however, given the lower current densities found in tissue not directly stimulated by tDCS, that non-neuronal modulation could explain tDCS-induced BOLD signal changes at distant sites.

In addition to the potential ambiguity for interpreting BOLD signal changes due to the effects of tDCS, vascular changes following stroke also complicate interpretation of BOLD signal changes in this population, especially within the hemisphere ipsilesional to the stroke. Assumptions regarding neurovascular coupling in patients, which are necessary for the analysis of BOLD data, may not be accurate in older patients with known abnormalities of blood supply (Blicher et al., 2012). It is likely that this potential confound is of most importance in the lesional and peri-lesional tissue – it is not clear to what extent these factors are important when interpreting activation changes in the contralesional hemisphere in stroke patients.

Despite these caveats, however, BOLD fMRI remains the mainstay for investigating tDCS-induced changes in brain activity in patients in the chronic stages of stroke recovery as it is widely available and has relatively good signal-to-noise, allowing for clinically feasible acquisition times and better temporal and spatial resolution than many other newly emerging techniques such as arterial spin labeling (ASL).

## Cortical and Subcortical Effects of tDCS

Modeling studies suggest that the regions of highest current density, and therefore the maximal direct effect of tDCS, are localized close to the stimulating electrodes, often relatively deep within the gyri (Miranda et al., 2006). However, there is increasing evidence that, in addition to local effects, tDCS modulates spinal excitability in humans (Roche et al., 2009), a finding supported by recent animal studies showing effects on subcortical structures (Bolzoni et al., 2013a,b). It is not clear whether these effects are mediated directly, or via excitability changes in the stimulated cortex, but such effects should be borne in mind when studying behavioral effects of stimulation.

## A Theoretical Framework for Understanding Stroke Recovery

One of the commonest models to explain the recovery of function after a motor stroke is that of inter-hemispheric imbalance. Robust imaging evidence from a number of studies suggests that after a stroke there is increased activity within the primary motor cortex of the contralesional hemisphere when the patient moves their stroke-affected hand (Chollet et al., 1991; Weiller et al., 1992, 1993; Calautti and Baron, 2003). This increased activation is greater in patients who make a poor functional recovery (Ward et al., 2003b) and longitudinal studies have demonstrated that it decreases over time in line with functional recovery (Ward et al., 2003a), such that patients who make a better recovery show less activation in this region. In addition, a study of nine patients who were in the chronic stages of stroke recovery and who had made a relatively poor recovery had increased inhibition from the contralesional M1 to the ipsilesional M1 (Murase et al., 2004). Although it is not clear whether this increase in inter-hemispheric inhibition is a direct reflection of recovery processes or disuse of the affected limb, taken together, these findings have informed the hypothesis that increased activity in the contralesional hemisphere is maladaptive to recovery.

Two major targets for neuromodulation in stroke rehabilitation have therefore emerged: it has been utilized either as a tool to directly increase activity within the ipsilesional motor cortex (M1_Ipsi_) or to decrease activity within the contralesional M1 (M1_Cont_) and thereby indirectly increase activity within M1_Ipsi_. Anodal tDCS applied to M1_Ipsi_ (Hummel et al., 2005, 2006; Stagg et al., 2012) and cathodal tDCS to the M1_Cont_ (Fregni et al., 2005; Bradnam et al., 2012), in both cases with the reference electrode over the contralateral supraorbital ridge, have been trialed in single sessions in patients in the chronic stage of stroke recovery with some evidence of short-lived behavioral improvements. In addition, a “bihemispheric montage,” whereby the anode was placed on M1_Ipsi_ and the cathode over M1_Cont_, has shown promise in a recent study (Lindenberg et al., 2010b).

However, there is also divergent evidence suggesting that activity within the contralesional hemisphere, both within M1_Cont_ and in the premotor cortices, may also be *adaptive* in at least some patients (Johansen-Berg et al., 2002; Gerloff et al., 2006; Lotze et al., 2006). Here, we will review the evidence from the imaging literature supporting the theory that tDCS can modulate inter-hemispheric imbalance before evaluating the evidence for this modulation underlying the behavioral improvements seen after tDCS in stroke patients.

## Single Session fMRI Studies

### Studies in healthy controls

A number of imaging studies in healthy controls have investigated the effects of tDCS on motor-related activity (Baudewig et al., 2001; Kwon et al., 2008; Stagg et al., 2009b; Lindenberg et al., 2013). Of these, one study has utilized “standard” stimulation parameters (Stagg et al., 2009b) and suggested that anodal tDCS applied to the left M1 during a simple finger-tapping task performed with the right hand led to an increase in movement-related BOLD signal under the stimulating electrode and in anatomically connected regions within the stimulated hemisphere (Stagg et al., 2009b). Cathodal tDCS applied to the left M1 during the same task also led to an increase in BOLD signal under the stimulating electrode. In addition, however, there was an increase in movement-related signal in the contralateral, unstimulated, hemisphere after cathodal tDCS, a finding in line with the inter-hemispheric imbalance hypothesis that decreasing activity in one M1 leads to increased activity in the contralateral hemisphere. This finding is supported by an earlier transcranial magnetic stimulation (TMS) study suggesting that cathodal tDCS decreases the duration of transcallosal inhibition from the stimulated hemisphere (Lang et al., 2004).

Another study tested the effects of a conventional unilateral montage as well as the bihemispheric montage (also described as “dual” stimulation), described above, where the anode is positioned over one M1 and the cathode over the opposite M1 (Lindenberg et al., 2013). Older adults performed a choice reaction time task during stimulation. The main finding of the study was that greater activity in bilateral M1 was observed during bihemispheric tDCS compared to unilateral anodal tDCS. This is consistent with a simple minded prediction that could be made by combining the unilateral effects described in the study by Stagg and colleagues above. However, in contrast to the results from the Stagg and colleagues study, unilateral anodal tDCS was not found to have any effect on task related activity. These two studies differ in many important respects that could explain this difference in findings. In particular, the study by Lindenberg and colleagues included older participants, used a complex motor task, and acquired fMRI data during, rather than after, tDCS.

#### Studies of resting state activity

Transcranial direct-current stimulation has been demonstrated in a number of studies to modulate resting connectivity across the brain, although to date no clear consensus across the literature has emerged as to the specific pattern of stimulation-induced changes (Polanía et al., 2011, 2012a,b; Zheng et al., 2011; Sehm et al., 2012, 2013; Amadi et al., 2013; Stagg et al., 2013). This lack of agreement between studies as to the effects of tDCS most likely reflects differences in MR acquisition and stimulation parameters, as well as the likely sensitivities of different analysis approaches, but makes interpretation of the literature as it stands somewhat problematic.

### Studies in chronic stroke patients

It is not clear whether findings in healthy controls can be extrapolated to stroke patients, in whom brain damage and subsequent plasticity might alter responses to tDCS. We therefore recently tested the effects of tDCS on fMRI signals in a cohort of chronic stroke patients (Stagg et al., 2012). Patients were asked to perform a simple visually cued reaction time task with their paretic limb during fMRI acquisition, before and after 10 min of 1 mA tDCS. The study compared the effects of anodal tDCS applied to the ipsilesional M1 (M1_Ipsi_) and cathodal tDCS applied to the contralesional M1 (M1_Cont_) to a sham (placebo) tDCS condition. Reaction times were used as an outcome measure in this study as simple measures of behavior are required for fMRI studies. Although not a clinical outcome, reaction time has frequently been used as a surrogate outcome measure in tDCS studies (Hummel et al., 2006; Stagg et al., 2012).

Behaviorally, anodal tDCS applied to M1_Ipsi_ led to an improvement in reaction times (Stagg et al., 2012), in line with previous reports of a functional benefit of this tDCS montage (Fregni et al., 2005; Hummel et al., 2005, 2006). In our cohort, however, cathodal tDCS applied to M1_Cont_ did not lead to a functional improvement, a finding at odds with a previous tDCS study (Fregni et al., 2005) and some studies of low frequency rTMS, which is also known to have an inhibitory effect on the stimulated cortex (Mansur et al., 2005; Takeuchi et al., 2005; Fregni et al., 2006a).

**Figure 1:**
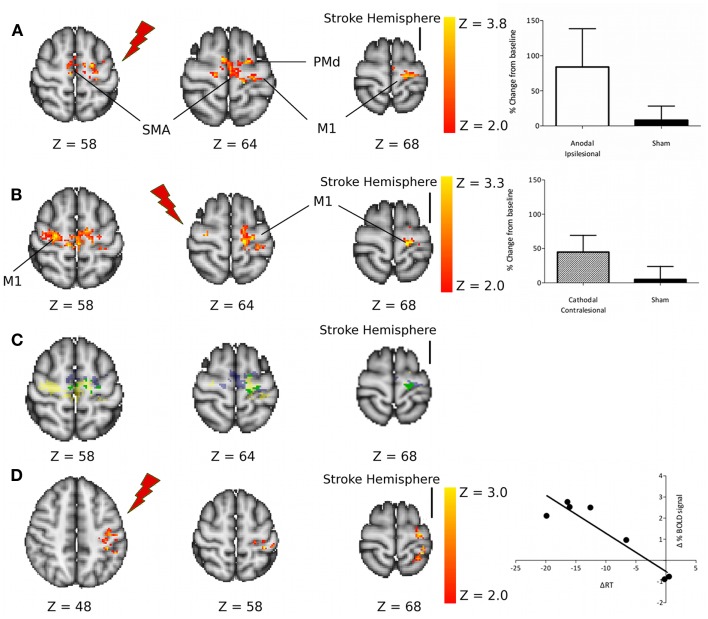
**(A)** Regions of increased motor-related activation after anodal stimulation applied to the ipsilesional M1 compared with sham. The column graph (top right) shows the mean change in activity within these regions. **(B)** Regions of increased motor-related activation after cathodal stimulation applied to the contralesional M1 compared with sham. The column graph (center right) shows the mean change in activity within these regions. **(C)** Regions of increased motor-related activation after M1_Ipsi_ anodal tDCS (blue), M1_Cont_ Cathodal tDCS (yellow), and regions where these two effects overlap (green). **(D)** Regions of significant correlation between the change in motor-related fMRI signal due to M1_Ipsi_ anodal tDCS and the tDCS-induced change in reaction times. M1, primary motor cortex; SMA, supplementary motor area; PMd, dorsal premotor cortex. Reprinted with permission from Figure 3, Stagg et al. (2012).

However, despite this important difference in behavioral effects between the two tDCS conditions, there were no significant differences between the two tDCS approaches in terms of their effects on movement-related fMRI activity. Both anodal tDCS to M1_Ipsi_ and cathodal tDCS to M1_Cont_ led to an increase in movement-related fMRI signal within the ipsilesional M1 in chronic stroke patients (Figure 1).

Taken together these results therefore suggest that tDCS is capable of modulating the functional interactions between the two motor cortices as hypothesized in the standard model of inter-hemispheric imbalance, as discussed above. However, these results also suggest that although down-regulation of the contralesional hemisphere (by cathodal tDCS to M1_Cont_) can lead to an increase in activity within the ipsilesional hemisphere, this alone is not sufficient to induce behavioral improvements in patients. There are two possible reasons for this: it may be that some patients rely on activity within their contralesional M1 to support their function in the paretic hand or it may be that direct modulation of activity within the ipsilesional M1 is necessary for a behavioral improvement to occur.

It is not possible to disambiguate these two possibilities from the data currently available, and indeed both these factors may play a role. Patients in our study were on average more impaired than those in a previous study showing positive behavioral effects of cathodal tDCS to M1_Cont_ in chronic stroke (Fregni et al., 2005), and an increasing number of studies suggest that, particularly in poorly recovered patients, activity in the contralesional hemisphere may be functionally adaptive (Johansen-Berg et al., 2002; Gerloff et al., 2006; Lotze et al., 2006; Bestmann et al., 2010).

**Figure 2:**
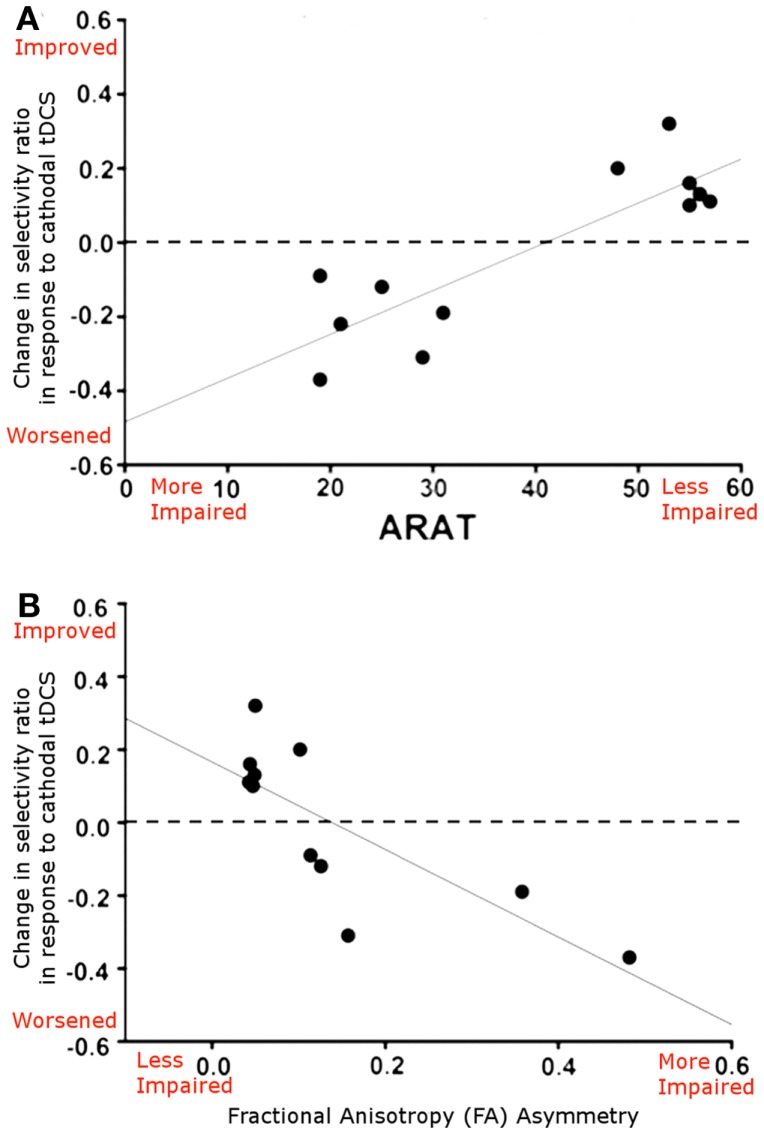
**(A)** Relationship between the neurophysiological response to cathodal tDCS applied to the contralesional M1 (reported as selectivity ratio, where higher numbers reflect better responses) and ARAT score, a measure of functional impairment, where higher numbers reflect better functioning. Patients who are better recovered show a beneficial functional response to cathodal tDCS applied to M1_Cont_, whereas those who are more poorly recovered show a neurophysiological worsening. **(B)** Scatterplot showing the relationship between the neurophysiological response to cathodal tDCS and structural integrity of the corticospinal tracts (reported as FA asymmetry, where higher values represent greater ipsilesional tract disruption). A clear relationship is demonstrated, whereby cathodal tDCS applied M1_Cont_ leads to a neurophysiological improvement in patients with good ipsilesional corticospinal tract integrity. Adapted from Figure 5, Bradnam et al. (2012).

In support of this argument, a recent paper by Bradnam et al. (2012) suggested that the neurophysiological effects of cathodal tDCS applied to M1_Cont_ were dependent on the level of patient’s functional recovery. In well-recovered patients cathodal tDCS to M1_Cont_ led to an improvement in neurophysiological measures whereas in more poorly recovered patients it led to a neurophysiological impairment. In addition, the authors acquired DTI data, which demonstrated that this dichotomy in effects was dependent on the integrity of the *ipsilesional* corticospinal tract (CST; Figure 2). This finding suggests that, although the contralesional M1 may indeed play an important role in behavioral recovery in the least well-recovered patients, the mechanism by which it exerts its effect is complex and likely involves an interplay between the contralesional and ipsilesional hemispheres.

In addition to this potential functional role of the contralesional M1, there is also evidence that the effects of tDCS on the directly stimulated cortex may be particularly important in determining the functional effects of tDCS after stroke.

#### Evidence for the importance of increased activity within M1_Ipsi_ for functional improvements

In our recent fMRI study in chronic stroke patients (Stagg et al., 2012), we performed a whole-brain analysis investigating whether the magnitude of the functional improvement induced by tDCS correlated with the magnitude of the change in the movement-related BOLD signal, to address the question of where within the brain a local increase in fMRI activity could be directly related to a behavioral improvement. A relationship between change in BOLD signal and reaction times was only demonstrated within the M1 and primary sensory cortex in the ipsilesional hemisphere (Figure 1D), and only for anodal tDCS (Stagg et al., 2012). This suggests that the degree of activity within M1_Ipsi_ is important in determining functional response. No relationship between BOLD signal modulation and RT change were demonstrated for cathodal tDCS applied to M1_Cont_, suggesting that the direct effects of anodal tDCS on M1_Ipsi_ may be important.

To date there has only been one published study investigating the effects of repeated sessions of tDCS in stroke patients that has utilized fMRI. Lindenberg et al. (2010b) studied the effects of five consecutive days of 30 min of 1.5 mA of bihemispheric tDCS combined with simultaneous physical/occupational therapy in patients in the chronic stages of recovery post motor stroke. They demonstrated that, compared with a placebo control, bihemispheric tDCS led to a functional improvement that was sustained at 1 week post intervention. The authors acquired fMRI data before and after the intervention and demonstrated using a whole-brain analysis that patients in the active treatment group showed an increase in fMRI activity within M1_Ipsi_. No significant change in fMRI activity was demonstrated in the placebo group, but the degree of fMRI change was not directly compared between the two groups.

The authors additionally demonstrated a significant positive correlation between the degree of improvement in their clinical outcome score (the Wolf Motor Function Test) and the change in laterality index (LI) of the fMRI signal within the pre-central gyri (Lindenberg et al., 2010b), suggesting that patients who showed greatest improvements in behavior were those in whom the balance of M1 activity had shifted most toward M1_Ipsi_.

The whole-brain analyses from this study support the suggestion that local increases in M1_Ipsi_ activity are the important factor in driving behavioral improvements. However, it is not possible from the LI data presented in the paper to know whether the relationship between LI and behavioral improvement was driven by a relationship between increased activity within M1_Ipsi_ or a true change in the balance of activity between the two motor cortices.

#### Evidence for the importance of local effects of tDCS

If the direct up-regulation of M1_Ipsi_ is important in functional gains post-stroke, can MR studies inform our understanding of what processes may underlie this functional improvement?

Magnetic resonance spectroscopy studies have begun to address this question in healthy controls, although at the time of writing no study has directly addressed the question in patients. In control subjects, anodal tDCS leads to a significant decrease in GABA within the stimulated cortex (Stagg et al., 2009a, 2011a), and GABA levels within M1 were shown to be closely related both to reaction times and to movement-related fMRI signal (Stagg et al., 2011a). GABA levels (as assessed by TMS) have previously been shown to be decreased in chronic stroke patients (Hummel et al., 2009); plasticity within the primary motor cortex is critically dependent on GABAergic modulation (Hess et al., 1996; Trepel and Racine, 2000); and application of a GABA antagonist in animal models leads to a significant improvement in recovery (Clarkson et al., 2010).

The role of GABA in plasticity and recovery supports the theory that direct application of anodal tDCS to M1_Ispi_ may lead to behavioral improvements directly by modulating local inhibitory tone, something that would not necessarily occur with cathodal tDCS applied to M1_Cont_. However, this hypothesis has yet to be directly tested and should not be taken to rule out the probability that glutamatergic processes, which are more challenging to measure using MRS, are also important for local plasticity within M1.

## Conclusion

In this review we have summarized the evidence from MR studies supporting the use of tDCS in the functional recovery from stroke. With the increased availability of high-field MR systems and technical advances meaning that many advanced MR techniques are becoming possible within a clinically feasible timescale, the utility of MR to inform our understanding of the effects of neuromodulation is increasing. Furthermore, there is a growing case for using MR measures, in combination with other techniques such as TMS, and clinical scores to stratify tDCS interventions on a patient-by-patient basis (Stinear et al., 2007, 2012; Bradnam et al., 2011).

In particular, understanding the importance of using cathodal tDCS to down-regulate activity within the contralesional hemisphere and the importance of direct, rather than indirect, up-regulation of activity within M1_Ipsi_ are likely to be of great importance to maximizing the utility of tDCS on a patient-by-patient basis. This is most likely to be achieved by the increasing use of a combination of clinical scores, TMS, and MR measures to provide a powerful way to optimize therapeutic interventions. It is likely that the exact combination of measures that gives the optimal power for stratification will depend on which muscle groups are being studied.

Transcranial direct-current stimulation is a promising clinical tool for stroke rehabilitation (Butler et al., 2013) but, like much of the stroke rehabilitation literature (Stinear et al., 2013), studies investigating its use are small and their power to investigate how tDCS may be optimized on a patient-by-patient basis are therefore inherently limited. In order to fully explore the potential of the technique larger, probably necessarily multi-center, studies are required which are both properly controlled and adequately powered to study potential patient stratification.

## Conflict of Interest Statement

The authors declare that the research was conducted in the absence of any commercial or financial relationships that could be construed as a potential conflict of interest.
